# Assessment, management, and incidence of neonatal jaundice in healthy neonates cared for in primary care: a prospective cohort study

**DOI:** 10.1038/s41598-022-17933-2

**Published:** 2022-08-23

**Authors:** Berthe A. M. van der Geest, Malou J. S. de Mol, Ivana S. A. Barendse, Johanna P. de Graaf, Loes C. M. Bertens, Marten J. Poley, Erwin Ista, René F. Kornelisse, Irwin K. M. Reiss, Eric A. P. Steegers, Jasper V. Been, STARSHIP Study Group, Martin G. A. Baartmans, Jolita Bekhof, Harry Buijs, Jan Erik Bunt, Peter H. Dijk, Christian V. Hulzebos, Ralph W. J. Leunissen, Ben J. P. W. Snoeren, Bente de Vries, Leo Wewerinke

**Affiliations:** 1 Division of Neonatology, Department of Paediatrics, Erasmus MC–Sophia Children’s Hospital, University Medical Centre Rotterdam, Rotterdam, The Netherlands; 2 Division of Obstetrics and Fetal Medicine, Department of Obstetrics and Gynaecology, Erasmus MC, University Medical Centre Rotterdam, Rotterdam, The Netherlands; 3 Institute for Medical Technology Assessment (iMTA), Erasmus University Rotterdam, Rotterdam, The Netherlands; 4 Intensive Care and Department of Paediatric Surgery, Erasmus MC–Sophia Children’s Hospital, University Medical Centre Rotterdam, Rotterdam, The Netherlands; 5 Department of Paediatrics, Intensive Care Unit, Erasmus MC–Sophia Children’s Hospital, University Medical Centre Rotterdam, Rotterdam, The Netherlands; 6 Department of Internal Medicine, Nursing Science, Erasmus MC, University Medical Centre Rotterdam, Rotterdam, The Netherlands; 7 Department of Public Health, Erasmus MC, University Medical Centre Rotterdam, Rotterdam, The Netherlands; 8 Department of Paediatrics, Maasstad Hospital, Rotterdam, The Netherlands; 9 Department of Paediatrics, Isala–Amalia Children’s Clinic, Zwolle, The Netherlands; 10 Primary Care Birth Centre Haga, The Hague, The Netherlands; 11 Primary Care Birth Centre Maasstad, Rotterdam, The Netherlands; 12 Department of Paediatrics, Elisabeth-TweeSteden Hospital, Tilburg, The Netherlands; 13 Department of Neonatology, University Medical Centre Groningen–Beatrix Children’s Hospital, University of Groningen, Groningen, The Netherlands; 14 Department of Paediatrics, Haaglanden Medical Centre Westeinde, The Hague, The Netherlands; 15 Primary Care Birth Centre Fam, Tilburg, The Netherlands; 16 Primary Care Birth Centre Westeinde, The Hague, The Netherlands; 17 Department of Paediatrics, Haga Hospital–Juliana Children’s Hospital, The Hague, The Netherlands

**Keywords:** Paediatric research, Neonatology

## Abstract

Jaundice caused by hyperbilirubinaemia is a common phenomenon during the neonatal period. Population-based studies evaluating assessment, management, and incidence of jaundice and need for phototherapy among otherwise healthy neonates are scarce. We prospectively explored these aspects in a primary care setting via assessing care as usual during the control phase of a stepped wedge cluster randomised controlled trial.

We conducted a prospective cohort study embedded in the Screening and TreAtment to Reduce Severe Hyperbilirubinaemia in Infants in Primary care (STARSHIP) Trial. Healthy neonates were included in seven primary care birth centres (PCBCs) in the Netherlands between July 2018 and March 2020. Neonates were eligible for inclusion if their gestational age was ≥ 35 weeks, they were admitted in a PCBC for at least  2 days during the first week of life, and if they did not previously receive phototherapy. Outcomes were the findings of visual assessment to detect jaundice, jaundice incidence and management, and the need for phototherapy treatment in the primary care setting.

860 neonates were included of whom 608 (71.9%) were visibly jaundiced at some point during admission in the PCBC, with 20 being ‘very yellow’. Of the latter, four (20%) did not receive total serum bilirubin (TSB) quantification. TSB levels were not associated with the degree of visible jaundice (p = 0.416). Thirty-one neonates (3.6%) received phototherapy and none received an exchange transfusion. Five neonates did not receive phototherapy despite having a TSB level above phototherapy threshold.

Jaundice is common in otherwise healthy neonates cared for in primary care. TSB quantification was not always performed in very jaundiced neonates, and not all neonates received phototherapy when indicated. Quality improvement initiatives are required, including alternative approaches to identifying potentially severe hyperbilirubinaemia.

**Trial registration:** NL6997 (Dutch Trial Register; Old NTR ID 7187), registered 3 May 2018.

## Introduction

Neonatal hyperbilirubinaemia is a common condition during the first days of life and typically presents as visible jaundice^1^. Hyperbilirubinaemia in the neonatal period is usually benign. In some neonates, unconjugated bilirubin may reach hazardous levels and cause acute bilirubin encephalopathy and later kernicterus spectrum disorder (KSD) when not timely recognised and treated^2^.

In several countries and settings, the first-line recognition of hyperbilirubinaemia is based on visual inspection of jaundice, followed by selective total serum bilirubin (TSB) quantification (i.e., if considered necessary). Transcutaneous bilirubin quantification is not widely used in the primary care setting. TSB levels are plotted on a nomogram to determine the need for treatment (Text Box 1)^3–5^. Phototherapy is a safe and effective treatment to decrease bilirubin levels and is usually applied in-hospital^1^. When bilirubin levels are extremely high or continue to increase despite intensive phototherapy, one or more exchange transfusions may be needed to decrease bilirubin levels.

Although neonatal jaundice is commonly observed, population-based data on the assessment, management, and incidence of visual jaundice and need for phototherapy among healthy neonates, especially if cared for in primary care, are scarce. Whereas the inaccuracy of visual inspection of jaundice to estimate TSB levels has previously been demonstrated^6,7^, the associations of visual jaundice assessment to the decision whether or not to quantify TSB, and of visual jaundice assessment to whether or not a neonate exceeded the individual phototherapy treatment threshold in primary care are unknown. Also, most population-based studies focus on hospitalised neonates having severe neonatal hyperbilirubinaemia or KSD^8–11^. Hence, these studies do not cover the complete scope of assessment, management, incidence, and burden of neonatal hyperbilirubinaemia. In addition, definitions of severe hyperbilirubinaemia vary, resulting in a wide variation in reported incidences of neonatal hyperbilirubinaemia between studies^12–17^.

The Screening and TreAtment to Reduce Severe Hyperbilirubinaemia in Primary care (STARSHIP) Trial is an ongoing factorial stepped-wedge cluster randomised controlled trial in seven Dutch primary care birth centres (PCBCs). It aims to assess the effectiveness of universal transcutaneous bilirubin (TcB) screening and of phototherapy applied in primary care^18^. See Text Box 2. In each participating PCBC, the initial phase of the STARSHIP trial evaluates usual care (i.e. no interventions are implemented). This provides a unique opportunity to explore the assessment, management, and incidence of neonatal jaundice and phototherapy in primary care among children included during this initial phase.

## Methods

### Study design

Prospective cohort study embedded in the factorial stepped-wedge cluster randomised controlled STARSHIP Trial^18^.

### Setting

In the Netherlands, most healthy neonates are either born in primary care or discharged to primary care (i.e. the home or a PCBC) within the first few hours to days of life^19^. A maternity care assistant (MCA) provides postpartum care to mother and neonate during daytime for the first 8 days after delivery^20^. The MCA is supervised by a community midwife, who visits the family at least three times in the first week^21^. The MCA assesses each day whether the neonate is visually jaundiced and if so, to which degree. The MCA is expected to consult the community midwife if she considers the neonate ‘too jaundiced’ or if she feels that there are other reasons to quantify TSB. Medical doctors are only involved in the care of otherwise healthy neonates if consulted by the community midwife. The current national multidisciplinary guideline on neonatal hyperbilirubinaemia does not include universal screening, but alternatively states that each involved perinatal healthcare professional should be aware of a neonate’s a priori risk for developing hyperbilirubinaemia and that this risk should be documented and communicated among all involved perinatal healthcare professionals^4^. According to the guideline, the healthcare provider may decide to have blood taken to quantify TSB levels if hyperbilirubinaemia is suspected based on visual inspection (e.g., a neonate is assessed ‘too jaundiced’). The guideline does not provide objective criteria for having TSB quantified^4^. One of the PCBCs and a small number of primary care midwifery practices participating in the STARSHIP Trial used selective transcutaneous bilirubin (TcB) screening (i.e., TcB quantification if a neonate is assessed ‘too jaundiced’, followed by TSB quantification if the TcB level is above or < 50 µmol/L below the phototherapy threshold). TSB levels are plotted on the Dutch TSB nomogram (Text Box 1), which is based on the American Academy of Pediatrics guidelines^3^. A paediatrician of a nearby affiliated hospital can be consulted when hyperbilirubinaemia is confirmed, and this is then usually treated in the hospital.

The STARSHIP Trial is conducted in seven PCBCs throughout the Netherlands where MCAs provide postpartum care, supervised by community midwives. Women can choose to receive their care either at home or in a PCBC if the neonate is healthy. Neonates included in the control phase of the STARSHIP Trial, when usual care was evaluated, were included in this cohort. The control phase of the STARSHIP Trial ran between 2 July 2018 and 8 March 2020 (Supplementary Table 1)^18^.

**Figure 1:**
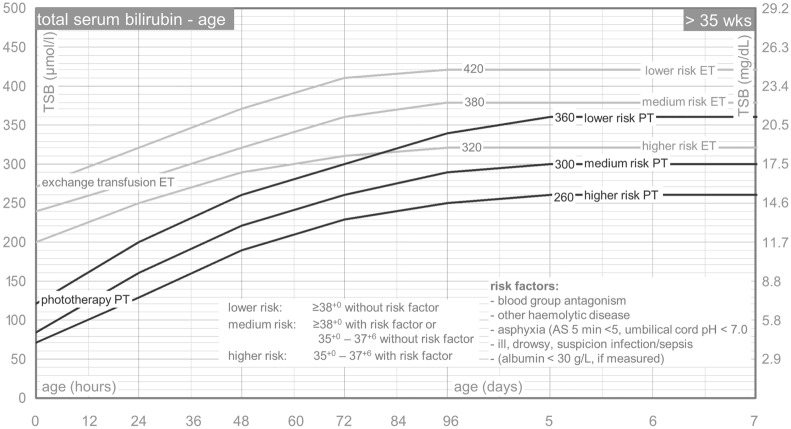
Phototherapy and exchange transfusion thresholds for neonates born after more than 35 weeks of gestation. *TSB* total serum bilirubin, *PT* phototherapy, *ET* exchange transfusion, *AS* Apgar score. Translated from Dutch. The Dutch nomogram is available at: http://babyzietgeel.nl/kinderarts/hulpmiddelen/diagnostiek/bilicurve35wkn.php.

Text Box 1: the Dutch TSB nomogram The Dutch TSB nomogram is adapted from the American Academy of Pediatrics^3^. Treatment thresholds are based on postnatal age and risk assessment. Gestational age (< 38 weeks or ≥ 38 weeks) and risk factors (blood group antagonism, haemolytic disease; birth asphyxia; suspicion of infection; drowsy or ill neonate; and serum albumin level below 30 g/L) are combined to assess the risk category: lower, medium, or higher risk. See Fig. 1.

### Participants

Neonates were eligible for inclusion in the STARSHIP Trial if:

- Born ≥ 35 + 0 weeks of gestation;
- Admitted to a participating PCBC during the first week of life;
- Expected to remain admitted to the PCBC for at least 2 days;
- Signed informed consent from parent(s) or primary caregiver(s) was obtained.

Neonates were not eligible if:

- The neonate previously received phototherapy;
- Parents did not have sufficient understanding of the Dutch language to be able to comprehend the patient information form.

For the analyses presented in this manuscript, all neonates included in the control phase of the STARSHIP Trial were eligible. Inclusion of the neonates was performed at admission to the PCBC and irrespective of the degree of jaundice of the neonate.

### Variables

Outcomes of this study are: findings of assessment of jaundice by MCAs (ranging from ‘not yellow at all’ to ‘very yellow’; in the Netherlands no standardised colour scale is used for visual jaundice assessment), the number of neonates in whom TSB was quantified; TSB level; management of neonatal hyperbilirubinaemia (i.e., what treatment is needed and what treatment is performed); the incidence of neonatal hyperbilirubinaemia and of receiving phototherapy treatment; and risk factors associated with receiving phototherapy. An overview of all variables used for the current analyses and definitions of variables is shown in Supplementary Table 2.

### Data sources

Baseline data regarding mother and neonate, and daily data regarding findings of screening and treatment of neonatal hyperbilirubinaemia were collected by MCAs of the participating PCBCs and by study personnel of the STARSHIP Trial and stored in a Limesurvey/Gemstracker database^22^. Additionally, parent(s) of all included neonates were asked to fill out a questionnaire, 2 weeks after discharge from the PCBC, that included questions regarding hospital admission for hyperbilirubinaemia. If a neonate was admitted to the hospital for neonatal hyperbilirubinaemia, additional information from the medical records regarding likely underlying causes, TSB levels, and treatment of hyperbilirubinaemia was requested from this hospital.

### Statistical analysis

Analyses were performed using SPSS Statistics version 25.0. Data were summarised using descriptive statistics. Mean and standard deviation (SD) were calculated for continuous, normally distributed data. For non-normally distributed data, median and interquartile range (IQR) were calculated. As phototherapy treatment thresholds vary according to postnatal age and individual risk assessment for each neonate (Text Box 1), the difference between a neonate’s TSB level and the corresponding phototherapy threshold for each individual neonate was calculated^4,5^. In the absence of information on individual risk factors determining phototherapy thresholds, such as blood group incompatibility, the risk factor is generally considered to be absent. To compare whether or not TSB was quantified, and the difference between neonates’ TSB levels and corresponding phototherapy thresholds among neonates having different degrees of visual jaundice, χ^2^ and Kruskal–Wallis test were performed as appropriate. Logistic regression was performed to analyse which risk factors were independently associated with hyperbilirubinaemia necessitating treatment. A p-value < 0.05 was considered to indicate statistical significance.

### Ethics

The STARSHIP Trial has been reviewed and approved by the Medical Research Ethics Committee of Erasmus MC Rotterdam, the Netherlands (MEC2017-473). The STARSHIP Trial was performed in accordance with the Declaration of Helsinki^23^.

### Consent to participate

Parents provided written informed consent before participation of their neonate in the study.

Text Box 2: STARSHIP Trial The Screening and TreAtment to Reduce Severe Hyperbilirubinaemia in Infants in Primary care (STARSHIP) Trial is a factorial stepped-wedge cluster randomised controlled trial. In the STARSHIP Trial, universal transcutaneous bilirubin screening and phototherapy in primary care are evaluated. The STARSHIP Trial is conducted in seven primary care birth centres (PCBCs) in the Netherlands. MCAs provide postpartum care supervised by community midwives in PCBCs. Medical doctors are not involved in providing care, although in some PCBCs they can be consulted if a problem arises.

According to the factorial stepped-wedge cluster design of the STARSHIP Trial, each PCBC is allocated to a predefined timeline with three phases. Each PCBC starts with a control phase in which all included neonates receive standard care according to the national multidisciplinary hyperbilirubinaemia guideline (i.e., visual inspection of jaundice and selected TSB quantification to screen for hyperbilirubinaemia, and phototherapy in the hospital if treatment is indicated)^4^. The control phase is followed by a second phase in which one intervention is implemented (i.e., transcutaneous bilirubin screening *or* phototherapy in the PCBC rather than in-hospital) and eventually by a final phase in which both interventions are implemented (i.e., transcutaneous bilirubin screening *and* phototherapy in the PCBC)^18^.

## Results

In total, 860 neonates were included in the control phase of the STARSHIP Trial. Baseline characteristics are shown in Table 1. Median gestational age was 39.3 weeks (IQR 1.9) and mean birth weight was 3399 g (SD 487). Most neonates were born after a vaginal, non-instrumental delivery, had a Western ethnicity and a Rh D positive mother. Apgar score at 5 min was below 5 in 18 neonates (2.1%) and umbilical cord pH was below 7.0 in 11 (2.5%) out of 441 neonates in whom umbilical cord pH was quantified.

**Table 1:** Baseline characteristics.

		n = 860
**Sex**		
Female	n (%)	398 (46.7)
Male	n (%)	454 (53.3)
Missing	n	8
**Gestational age (weeks)**	Median (IQR)	39.3 (1.9)
Missing	n	10
**Birth weight (grams)**	Mean (SD)	3399 (487)
Missing	n	8
**Mode of delivery**		
Vaginal, non-instrumental	n (%)	477 (56.1)
Vaginal, instrumental	n (%)	68 (8.0)
C-section, non-instrumental	n (%)	298 (35.0)
C-section, instrumental^a^	n (%)	8 (0.9)
Missing	n	9
**Apgar score < 5 at 5 min**	n (%)	18 (2.2)
Missing or unknown	n (%)	24
**Umbilical cord pH quantified**	n (%)	441 (64.7)
Of which, umbilical cord pH < 7.0	n (%)	11 (2.5)
Umbilical cord pH not quantified	n (%)	241 (35.3)
Missing or unknown	n	178
**Maternal Rh D negative**	n (%)	119 (16.3)
Of which, fetal Rh D positive	n (%)	42 (35.3)
Missing or unknown maternal Rh D	n	131
Non-western ethnicity neonate^b^	n (%)	200 (28.1)
Missing	n	149
**Type of feeding**		
Exclusive breastfeeding	n (%)	533 (62.6)
Exclusive formula feeding	n (%)	176 (20.7)
Combination	n (%)	143 (16.8)
Missing	n	8
**PCBC**^**c**^		
Fam, Tilburg	n (%)	56 (6.5)
Haga, The Hague	n (%)	98 (11.4)
Isala, Zwolle	n (%)	207 (24.1)
Maasstad, Rotterdam	n (%)	219 (25.5)
Noord, Rotterdam	n (%)	83 (9.7)
Sophia, Rotterdam	n (%)	187 (21.7)
Westeinde, The Hague	n (%)	10 (1.2)

*SD* standard deviation, *IQR* interquartile range, *PCBC* primary care birth centre.

^a^C-section, instrumental refers to (1) a vaginally, instrumental delivery that failed and subsequently a C-section was performed or (2) the use of vacuum extraction or forceps during C-section to assist the delivery of the neonate’s head.

^b^According to the definition of Statistics Netherlands^24^.

^c^Duration of inclusion period differed per PCBC. See Supplementary Table 1.

### Assessment and incidence of neonatal hyperbilirubinaemia

The majority of neonates (n = 608, 71.9%) had some degree of jaundice at any point during admission in the PCBC; the maximum degree of jaundice was ‘slightly yellow’ in the vast majority of jaundiced neonates (n = 442, 72.7%). In most neonates, jaundice was first noted on postnatal day one or two (n = 390, 75.0% of neonates having some degree of jaundice); two neonates (0.3%) were jaundiced within 24 h after birth (i.e., on postnatal day 0). TSB was quantified at least once in 129 neonates (15.0%). Twenty-three neonates (2.7%) had a TSB level above the phototherapy threshold during PCBC admission, at a median age of 57 h (IQR 43)^4^. In an additional five neonates, TSB level was above phototherapy threshold after discharge home from the PCBC (postnatal age range: 40–142 h), see Table 2.

**Table 2:** Assessment and incidence of neonatal hyperbilirubinaemia.

		n = 860
**Neonates having any degree of jaundice as assessed by MCA; maximum degree**	n (%)	608 (71.9)
Slightly yellow	n (%)	442 (72.7)
Moderately yellow	n (%)	91 (15.0)
Quite yellow	n (%)	61 (10.0)
Very yellow	n (%)	14 (2.3)
Missing visual jaundice assessment	n	14
**First postnatal day of jaundice during admission in PCBC (n = 608)**^**a**^		
Day 0 (0–23 h)	n (%)	2 (0.9)
Day 1–2 (24–71 h)	n (%)	390 (75.0)
Day 3–5 (72–143 h)	n (%)	202 (73.2)
Day 6–8 (144–215 h)	n (%)	8 (23.5)
Missing first day of jaundice	n	6
**Neonates who had TcB quantified in PCBC**	n (%)	116 (13.5)
1 TcB quantification	n (%)	80 (9.3)
2 or more TcB quantifications	n (%)	36 (4.2)
**Neonates who had TSB quantified in PCBC (before start of phototherapy, if indicated)**	n (%)	129 (15.0)
1 TSB quantification	n (%)	96 (11.1)
2 or more TSB quantifications	n (%)	33 (3.8)
**Bilirubin nomogram risk category**		
Lower risk	n (%)	664 (77.2)
Medium risk	n (%)	172 (20.0)
Higher risk	n (%)	14 (1.6)
Missing	n	10
**Highest TSB level during admission in PCBC (µmol/L; n = 124)**	Mean (SD)	223 (68)
TSB level missing	n	5
**Neonates having a TSB level above phototherapy threshold during admission in PCBC**^**b**^	n (%)	26 (3.0)
TSB level missing	n	2
**Postnatal age when exceeding phototherapy threshold in PCBC (hours; n = 23)**	Median (IQR)	57 (43)
**Neonates having a TSB level above phototherapy threshold after discharge home**	n (%)	7 (0.8)
Missing	n	148

*MCA* maternity care assistant, *PCBC* primary care birth centre, *TcB* transcutaneous bilirubin, *TSB* total serum bilirubin, *SD* standard deviation, *IQR* interquartile range.

^a^Percentage according to number of participating neonates that had some degree of jaundice during admission in the PCBC and were admitted in a participating PCBC at the time.

^b^Phototherapy threshold according to the Dutch TSB nomogram^4,5^.

**Figure 2:**
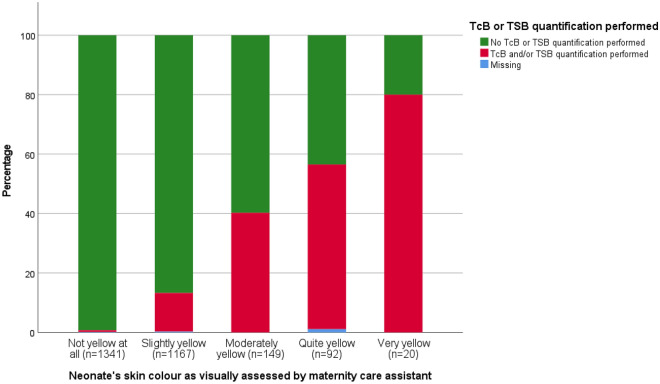
Proportion of assessment resulting in TcB or TSB being quantified according to degree of visible jaundice. *TcB* transcutaneous bilirubin, TSB total serum bilirubin.

In total, 165 TcB and 171 TSB quantifications were performed during admission in the PCBC. Figure 2 shows the association between visual jaundice assessment by the MCA and whether or not TcB or TSB quantification was performed. Although there was a clear increase in the proportion of neonates having TcB or TSB quantified as jaundice was considered more severe (χ^2^ trend test p < 0.001), still no TcB or TSB was quantified in 44% of the neonates considered ‘quite yellow’ and in 20% in of the neonates considered ‘very yellow’.

**Figure 3:**
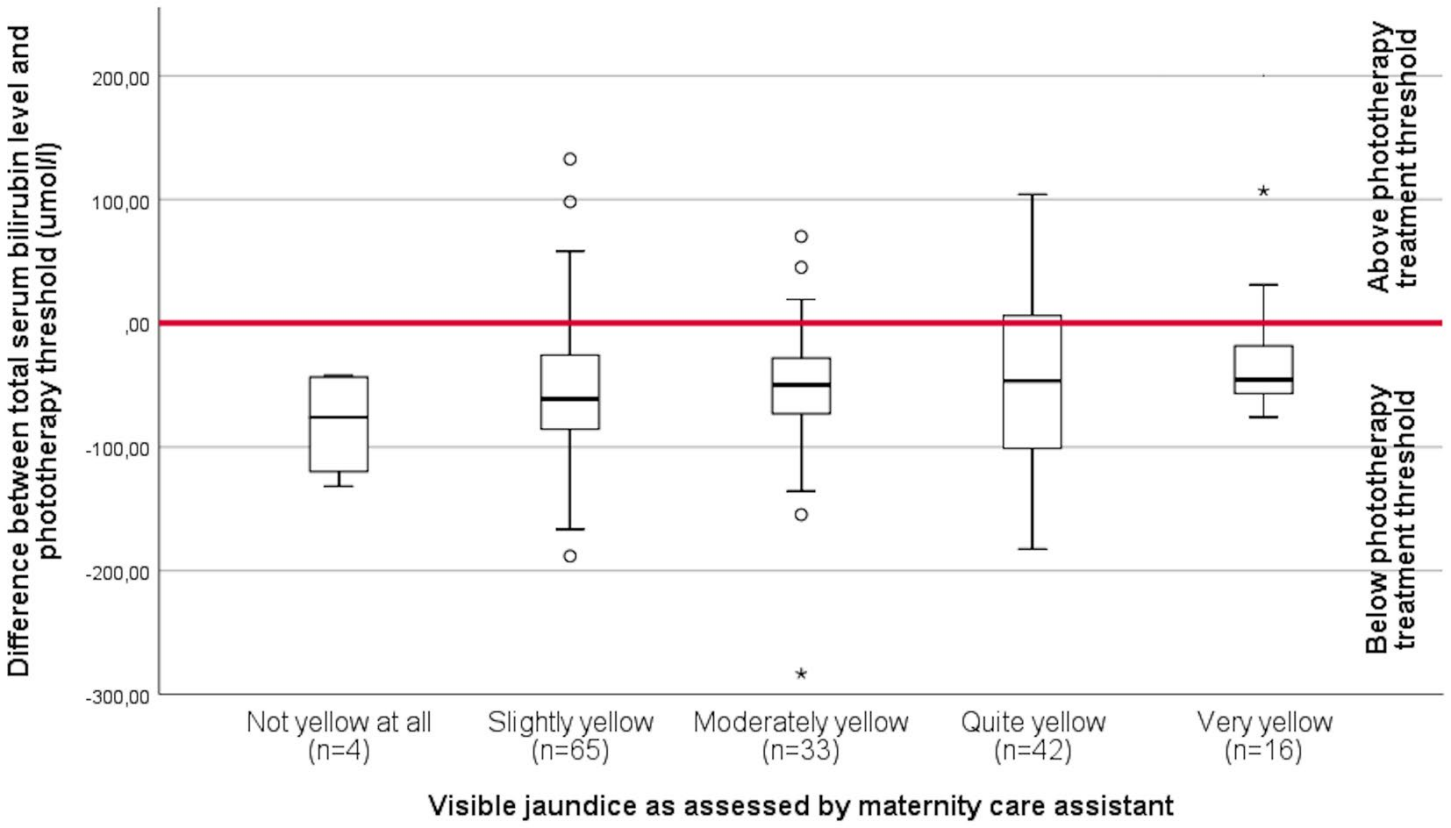
Difference between individual phototherapy treatment threshold and total serum bilirubin level according to degree of jaundice as visually assessed. The area above the red bar indicates a total serum bilirubin level above phototherapy treatment threshold.

The difference between individual phototherapy treatment thresholds and TSB levels according to the visually assessed degree of jaundice is shown in Fig. 3. TSB was below the treatment threshold for all four assessments resulting in TSB being quantified in the absence of jaundice. There was no clear association between the degree of jaundice and the TSB level in those having TSB quantified (p = 0.416).

### Management of hyperbilirubinaemia

Table 3 shows the management of hyperbilirubinaemia in neonates who received treatment. During the control period of the STARSHIP Trial, 33 neonates (3.8%) had a TSB level above the phototherapy treatment threshold^4^. Phototherapy was performed in 31 neonates (3.6%) with a median duration of 22 h (IQR 22.5). Three neonates (0.3%) received phototherapy despite having a TSB level below the phototherapy threshold, whereas five neonates (0.6%) did not receive phototherapy despite having a TSB level above the phototherapy threshold^4^. TSB levels of the latter five exceeded phototherapy threshold with a maximum of 31 µmol/L (1.81 mg/dL). One of these neonates was admitted to the hospital for another reason than hyperbilirubinaemia treatment. TSB levels exceeded the threshold for exchange transfusion (with a maximum of 71 µmol/L; 4.15 mg/dL) during admission in the PCBC in three neonates (0.3%) and during hospital admission in one additional neonate (0.1%)^4,5^, but no exchange transfusions were performed. The neonates with TSB levels that exceeded the exchange transfusion threshold during admission in the PCBC were slightly yellow (n = 2) and very yellow (n = 1). None of these neonates had a TSB quantified in the PCBC prior to exceeding the exchange transfusion threshold.

**Table 3:** Hyperbilirubinaemia management.

		n = 858^a^
Neonates having hyperbilirubinaemia above the treatment threshold^4,5^	n (%)	33 (3.8)
Highest TSB level overall if necessitating treatment (µmol/L)	Mean (SD)	318 (50)
Phototherapy performed	n (%)	31 (3.6)
Total duration of phototherapy (hours)	Median (IQR)	22 (22.5)
Missing duration of phototherapy	n	1
Exchange transfusion threshold exceeded^b^	n (%)	4 (0.5)
Exchange threshold exceeded in PCBC	n (%)	3 (0.3)
Exchange threshold exceeded in hospital	n (%)	1 (0.1)
Exchange transfusion performed	n (%)	0 (0.0)

*SD* standard deviation, *IQR* interquartile range.

^a^The need for phototherapy was unknown for two neonates, as daily measurements and parental questionnaire were not filled out. These neonates were excluded from these analyses.

^b^Phototherapy threshold according to the Dutch TSB nomogram^4,5^.

### Risk factors for receiving phototherapy treatment

Neonates who received phototherapy were more often born before 38 weeks of gestation when compared to neonates not receiving hyperbilirubinaemia treatment (56.7% vs. 12.8%; p < 0.001). The proportion of neonates born after an instrumental delivery was higher in the group receiving phototherapy than in the group not receiving phototherapy (26.7% vs. 8.3%; p = 0.004). Birth weight percentile^25^, perinatal asphyxia, Rh D incompatibility, type of feeding, sibling(s) who received phototherapy, and ethnicity were not significantly different between neonates who received phototherapy and those who did not (Table 4).

**Table 4:** Association of risk factors with receiving treatment for hyperbilirubinaemia.

	Received phototherapy (n = 31)	Did not receive phototherapy (n = 827)	Total (n = 858)^a^	p value
**Gestational age**				< 0.001*
< 38 weeks	17 (56.7)	105 (12.8)	122 (14.4)	
≥ 38 weeks	13 (43.3)	713 (87.2)	726 (85.6)	
Missing	1	9	10	
**Mode of delivery**				0.004*
Non-instrumental	22 (73.3)	751 (91.7)	773 (91.0)	
Instrumental	8 (26.7)	68 (8.3)	76 (9.0)	
Missing	1	8	9	
**Birth weight percentile**^25^				0.398
< p10	5 (16.7)	88 (10.8)	93 (11.0)	
p10–p90	25 (83.3)	647 (79.1)	672 (79.2)	
> p90	0 (0.0)	83 (10.1)	83 (9.8)	
Missing	1	9	10	
Presence of perinatal asphyxia*				0.191
Yes	2 (7.1)	27 (3.3)	29 (3.5)	
No	26 (92.9)	783 (96.7)	809 (96.5)	
Missing	3	17	20	
**Presence of Rh D incompatibility**				0.920
Yes	2 (7.1)	40 (6.2)	42 (6.2)	
No	26 (92.9)	608 (93.8)	634 (93.8)	
Missing or unknown	3	81	182	
**Type of feeding**				0.673
Exclusive breastfeeding	17 (56.7)	516 (62.9)	284 (34.5)	
Non-exclusive or no breastfeeding	13 (43.3)	304 (37.1)	540 (65.5)	
Missing	1	9	10	
**Sibling received hyperbilirubinaemia necessitating treatment**				0.964
Yes	4 (13.8)	33 (4.8)	37 (5.2)	
No	25 (86.2)	649 (95.2)	674 (94.8)	
Missing	2	145	147	
**Ethnicity neonate**				0.154
Western	22 (75.9)	489 (71.7)	511 (71.9)	
Non-Western	7 (24.1)	193 (28.3)	200 (28.1)	
Missing	2	147	149	

^a^The need for phototherapy was unknown for two neonates, as daily measurements and parental questionnaire were not filled out. These neonates were excluded from this analysis.

*Defined as Apgar score < 5 at 5 min and/or umbilical cord pH < 7.0. According to the definition of the Dutch TSB nomogram.

## Discussion

In our prospective cohort study evaluating the assessment, management, and incidence of neonatal hyperbilirubinaemia and the need for phototherapy among neonates cared for in primary care, we found that approximately 70% of neonates became jaundiced at any point during the first days of life and that 3.6% received treatment for hyperbilirubinaemia. However, not all neonates who had a TSB level that exceeded the phototherapy threshold received phototherapy. Also, TcB or TSB levels were not quantified in a substantial proportion of neonates assessed as moderately to severely jaundiced. Visual jaundice assessment was not reliable in estimating TSB levels.

To the best of our knowledge, this study is the first to prospectively describe the full scope of assessment, management, and corresponding incidence of hyperbilirubinaemia in otherwise healthy neonates cared for in primary care. This provides insight in the overall burden of neonatal hyperbilirubinaemia in primary care. We were able to identify neonates requiring phototherapy following discharge home by using parental questionnaires. Using parents as a source for data also has some pitfalls. First, if parents indicated that their neonate received phototherapy after discharge from the PCBC, this was not always in agreement with the actual data from the medical records in the hospital. Second, despite the prospective nature of the study, a proportion of included neonates had missing data, primarily due to missing parental questionnaires (17.3%). This may have led to an underestimation of the proportion of neonates who needed treatment. However, among the 711 (out of 860) neonates whose parents did respond, only five extra neonates who received treatment were identified using the questionnaires. Thus, we expect minimal influence of the missing data on this outcome. Neonates born after a C-section were overrepresented in our study (36% vs. 15% nationally)^26^, probably because their mothers were more likely to stay (longer) in the PCBC. As C-section is not known as a protective or risk factor for neonatal hyperbilirubinaemia, we expect negligible impact on our results. Additionally, the informed consent procedure may have induced selection (e.g., neonates whose parents refused participation in the STARSHIP Trial may have had other demographic characteristics). In contrast, overestimation of the proportion of neonates receiving hyperbilirubinaemia treatment in the whole population may have occurred as well. This is because we were dependent on parental consent for participation of their neonate in the STARSHIP trial and parents having a previous child with hyperbilirubinaemia may have been more likely to provide informed consent. Unfortunately, we were unable to assess the incidence of receiving phototherapy and associated risk factors (e.g., siblings having received phototherapy) among neonates without consent. Other findings may also have been influenced by the trial itself. Before the start of the STARSHIP Trial, all maternity care professionals were trained regarding neonatal hyperbilirubinaemia and study procedures. The training and the trial may have raised awareness on neonatal hyperbilirubinaemia, potentially resulting in a lower threshold to assess the neonate as jaundiced and to quantify TSB. From a clinical perspective, this can be considered a positive development in the context of preventing severe hyperbilirubinaemia.

The incidence of visible jaundice in our study is comparable to other studies in (near) term neonates in which 60–90% became jaundiced^27–29^. The finding that visual jaundice assessment is not reliable to estimate TSB levels is in line with other studies describing the inaccuracy of visual jaundice assessment^6,7^. Strikingly, in a substantial proportion of neonates being assessed as ‘quite yellow’ or ‘very yellow’, no TcB or TSB was quantified. This observation corresponds with a previous study among MCAs regarding neonatal hyperbilirubinaemia, which showed structural underestimation of TSB levels and common application of a so-called ‘wait-and-see approach’ in visibly jaundiced neonates^30^. Moreover, despite being strongly recommended by the national guideline^4^, TSB was not quantified in two neonates who developed visible jaundice within 24 h after birth. Also, five neonates did not receive phototherapy despite having a TSB level that exceeded the phototherapy threshold as defined by the national guideline^4^. Our evaluation of standard practice in this cohort highlights significant gaps in guideline application. In the current study, we did not prospectively explore the considerations underlying these decisions. Previous studies indicate that lack of knowledge on guideline recommendations^31,32^, and systematic underestimation of the severity of jaundice based on visual assessment likely contributed^30^. Other potential reasons for non-compliance may include a belief that the recommendations in the guideline do not reflect the best care for the neonate (e.g., a healthcare provider may consider the phototherapy thresholds too conservative as evidence on exact phototherapy thresholds is lacking^33^, and TSB quantification is avoided to keep the neonate in primary care), or practical challenges regarding feasibility of guideline compliance in daily practice. Research focused on these considerations may be useful to improve guideline adherence. Non-compliance to neonatal jaundice guidelines can have potentially severe consequences, as demonstrated by Rennie et al. in a Swedish study where KSD was (potentially) avoidable in 11 out of 13 neonates having KSD^9^. Additionally, a national audit indicated that non-compliance to the guideline was an important contributing factor to severe neonatal hyperbilirubinaemia in the Netherlands^34^.

Most studies assessing the burden of neonatal hyperbilirubinaemia focused on severe neonatal hyperbilirubinaemia or on KSD^12–17^. Studies assessing the hospitalisation rate for neonatal hyperbilirubinaemia showed incidences for hyperbilirubinaemia treatment ranging from 0.55 to 2.62%^35–39^. The retrospective nature of these studies in which the researchers depended on correct registration of the diagnosis of hyperbilirubinaemia may have contributed to the lower published incidence. Studies having phototherapy use as secondary outcome when assessing the institution of a bilirubin screening programme found (slightly) higher percentages in their control group (4.2–6.1%)^38–40^. The difference in the percentage of neonates necessitating hyperbilirubinaemia treatment between these studies and ours may also be attributed to other hyperbilirubinaemia assessment and management strategies. In the Netherlands, neonates are typically screened visually for neonatal hyperbilirubinaemia, followed by selective TSB quantification; universal TcB or TSB screening is not performed. Additionally, in the Netherlands a relatively high proportion of neonates are cared for in primary care shortly after birth, where transcutaneous bilirubinometers are not widely used yet. Our current evaluation of care-as-usual indicates that TcB or TSB is often not quantified even in neonates who were considered quite yellow or very yellow. As such, it is possible that some neonates requiring phototherapy were not identified. In other countries, most neonates remain admitted in the hospital for several days after birth and TcB or TSB is quantified before discharge^3,15^.

Potential risk factors for developing severe hyperbilirubinaemia have been widely investigated^3,33^. Whereas gestational age < 38 weeks is a well-known risk factor, instrumental delivery itself is not widely investigated as risk factor^16,41–43^. Most studies focus on bruising and cephalic haematomas, that may arise from an instrumental delivery, which increases the risk for severe neonatal hyperbilirubinaemia^16,41^. Instrumental delivery may be a marker for another risk factor (e.g., large for gestational age; LGA)^44^. However, we did not find a higher LGA incidence in neonates receiving phototherapy. Other well-known risk factors for hyperbilirubinaemia, such as Rh D incompatibility, previous siblings who received phototherapy, and exclusive breastfeeding, did not differ significantly between neonates who received phototherapy and those who did not. This may in part be due to limited power.

Findings from this study are useful for perinatal healthcare providers in primary care as well as in secondary and tertiary care (e.g., if a neonate is admitted together with mother). Data on the incidence of jaundice and the need for hyperbilirubinaemia treatment can help raise awareness regarding the extent of the problem. This awareness should also include the inaccuracy of visual jaundice assessment. Although this inaccuracy has been demonstrated previously^29,45,46^, our current study indicates that many healthcare professionals still strongly rely on visual assessment, and this is in fact in line with the current Dutch guideline, which is now undergoing revision. Although not every case of severe hyperbilirubinaemia results in KSD, KSD is entirely preventable and should clearly be a never-event. As such, regarding severe hyperbilirubinaemia as a healthcare system failure may strengthen implementation of new strategies to prevent KSD^47^. More objective approaches to universal hyperbilirubinaemia screening, for example using a transcutaneous bilirubinometer, should be considered to improve early recognition of potentially severe hyperbilirubinaemia^3,48,49^. Even though the PCBCs and their healthcare professionals took part in a trial focused on hyperbilirubinaemia assessment and management, the recommendations of the national guideline regarding TSB quantification and start of phototherapy treatment were not adhered to in some cases. Hence, more knowledge regarding risk factors for hyperbilirubinaemia, when to quantify TSB, treatment thresholds, and adherence to the national guideline are important as well. Future research should focus on objective approaches of universal screening for potentially severe neonatal hyperbilirubinaemia in a primary care setting. The STARSHIP Trial will present results from implementing a universal screening programme in primary care using TcB in the next year or two.

In this prospective cohort study embedded in the STARSHIP Trial, assessment, management and incidence of neonatal jaundice and the need for phototherapy were evaluated. We demonstrated that the vast majority of neonates had some degree of jaundice during admission and that phototherapy was provided in 3.6% of neonates. Also, we showed that visual jaundice assessment was inaccurate in determining hyperbilirubinaemia and that compliance to the guideline requires improvement. We suggest that awareness regarding neonatal hyperbilirubinaemia and its potentially devastating consequences should be raised. Additionally, the benefits of objective universal screening to improve recognition of hyperbilirubinaemia need to be assessed in an attempt to reduce the burden of neonatal hyperbilirubinaemia.

## Supplementary Information

Supplementary Information.

## Data Availability

The anonymised datasets from the current study are available from the corresponding author on reasonable request.

## Code availability

The syntaxes from the current analyses are available from the corresponding author on reasonable request.
