# Supplementary material for: Assessment, management, and incidence of neonatal jaundice in healthy neonates cared for in primary care: a prospective cohort study
Source: Sci Rep. 2022 Aug 23;12:14385. doi: 10.1038/s41598-022-17933-2 (PMC9399078; doi:10.1038/s41598-022-17933-2)
Supplement: Supplementary file 1 — Supplementary Information. [file 41598_2022_17933_MOESM1_ESM.pdf]

SUPPLEMENTARY FILES: ASSESSMENT, MANAGEMENT, AND INCIDENCE OF NEONATAL JAUNDICE IN HEALTHY NEONATES CARED FOR IN PRIMARY CARE: A PROSPECTIVE COHORT STUDY – SCIENTIFIC REPORTS

Berthe A.M. van der Geest[1,2], Malou J.S. de Mol[1,2], Ivana S.A. Barendse[1,2], Johanna P. de Graaf[2], Loes C.M. Bertens[2], Marten J. Poley[3,4], Erwin Ista[5,6], René F. Kornelisse[1], Irwin K.M. Reiss[1], Eric A.P. Steegers[2], Jasper V. Been[1,2,7]*. On behalf of the STARSHIP study group.

[1]Department of Paediatrics, Division of Neonatology, Erasmus MC Sophia Children's Hospital, University Medical Centre Rotterdam, Rotterdam, the Netherlands.

[2]Department of Obstetrics and Gynaecology, Division of Obstetrics and Foetal Medicine, Erasmus MC, University Medical Centre Rotterdam, Rotterdam, the Netherlands.

[3]Institute for Medical Technology Assessment (iMTA), Erasmus University Rotterdam, Rotterdam, the Netherlands.

[4]Intensive Care and Department of Paediatric Surgery, Erasmus MC – Sophia Children's Hospital, University Medical Centre Rotterdam, Rotterdam, the Netherlands.

[5]Department of Paediatrics, Intensive Care Unit, Erasmus MC – Sophia Children's Hospital, University Medical Centre Rotterdam, Rotterdam, the Netherlands.

[6]Department of Internal Medicine, Nursing Science, Erasmus MC, University Medical Centre Rotterdam, Rotterdam, the Netherlands

[7]Department of Public Health, Erasmus MC, University Medical Centre Rotterdam, Rotterdam, the Netherlands.

*Corresponding author: Jasper V. Been, j.been@erasmusmc.nl.

SUPPLEMENTARY TABLE 1: START AND END DATES CONTROL PHASE STARSHIP TRIAL

PCBC	Start date control phase	End date control phase
Fam, Tilburg	21 November 2018	26 May 2019
Haga, The Hague	3 August 2018	12 May 2019
Isala, Zwolle	14 January 2019	8 March 2020
Maasstad, Rotterdam	13 September 2018	10 November 2019
Noord, Rotterdam	5 November 2018	1 May 2019
Sophia, Rotterdam	2 July 2018	14 April 2019
Westeinde, The Hague	23 January 2019	18 March 2019*

PCBC = primary care birth centre.

*The planned end date of the control phase in PCBC Westeinde was 10 July 2019. However, as of 18 March 2019 this PCBC was permanently closed due to unforeseen circumstances.

SUPPLEMENTARY TABLE 2: VARIABLES USED FOR ANALYSIS

Category	Variable
Maternal characteristics	Gestational age (days)
	Maternal birth country
	Maternal Rhesus D factor
	Parity
Family characteristics	Paternal birth country
	Siblings with history of neonatal hyperbilirubinaemia
Delivery characteristics	Mode of delivery (vaginal non-instrumental; vaginal, with vacuum; vaginal, with forceps C-section, non-instrumental; C-section, instrumental)
Neonatal characteristics at baseline	Sex (male; female; indistinct)
	Apgar score <5 after 5 minutes (no; yes; unknown)
	Arterial umbilical cord pH quantified (no; yes; unknown)<br>Arterial umbilical cord pH <7.0 (no; yes; unknown)
	Birth weight (grams)
	Type of feeding (multiple answers possible: Breastfeeding on demand; Breastfeeding on schedule; Mother's milk via bottle or finger feeding; Formula feeding)
	Foetal Rhesus D factor (not determined; Rhesus D positive; Rhesus D negative; unknown)
Daily measurements	Skin colour (not yellow at all; slightly yellow; moderately yellow; quite yellow; very yellow)
	Weight (grams)
	Risk factors for hyperbilirubinaemia (Blood group or Rhesus antagonism; Other haemolytic disorder; Asphyxia; Ill or drowsy neonate; Other, namely)
	TSB levels in µmol/L with age of neonate in hours at measurement (if relevant)

	Decisions made based on TSB (if relevant)
	Admission to hospital (no; yes)
Parental questionnaire	Hospital admission after admission in PCBC (yes; no)
Data requested from hospital (if relevant)	Duration of hospital admission in nights
	Duration of phototherapy (in hours)
	Exchange transfusion performed
	Number of exchange transfusions performed
	All laboratory quantifications during admission with age of neonate in hours at quantification
	Blood group and Rhesus D factor neonate
	Blood group and Rhesus D factor mother
	Risk factors for neonatal hyperbilirubinaemia (as described in medical records)
Variables composed for analysis	Any degree of jaundice: neonate having any degree of jaundice as assessed by MCA (i.e., slightly yellow, moderately yellow, quite yellow, or very yellow) during admission in PCBC.
	Maximum degree of jaundice: maximum intensity of jaundice of neonate during admission in PCBC.
	First postnatal day of visual jaundice during admission in PCBC: first day on which any degree of jaundice (i.e., slightly yellow, moderately yellow, quite yellow, or very yellow) was noted during admission in PCBC.
	Presence of perinatal asphyxia: Apgar score <5 at 5 minutes and/or umbilical cord pH <7.0*
	Exclusive breastfeeding: neonate was exclusively breastfed (i.e., no pumped mother's milk, no finger feeding).<br>Non-exclusive breastfeeding or formula feeding: neonate was fed with pumped mother's milk or formula feeding by bottle or finger feeding (sometimes in combination with being breastfed).
	Western neonatal ethnicity: mother and father (if known) are born in a Western birth country.<br>Non-Western neonatal ethnicity: mother or father (if known) are born in a non-Western birth country.#
	Age at discharge from the PCBC home or to the hospital: difference between discharge date and time and birth date and time in hours.<br>*If discharge date missing: day after last measurements in PCBC was considered as discharge date,*<br>*If discharge time was missing: 10.00h was considered as discharge time.*

TSB = total serum bilirubin; PCBC = primary care birth centre; MCA = maternity care assistant.

*As defined in the Dutch bilirubin nomogram.[1]

#According to the definition of Statistics the Netherlands.[2]

REFERENCES

1. [Paediatric Association of the Netherlands] Nederlandse Vereniging voor Kindergeneeskunde (2008) [Bilirubin nomograms >35 weeks] Bilicurve >35 wkn. [Paediatric Association of the Netherlands] Nederlandse Vereniging voor Kindergeneeskunde. http://babyzietgeel.nl/kinderarts/hulpmiddelen/diagnostiek/bilicurve35wkn.php.]
2. [Statistics Netherlands] Centraal Bureau voor de Statistiek (2016) Person with a western migration background. [Statistics Netherlands] Centraal Bureau voor de Statistiek. https://www.cbs.nl/en-gb/onze-diensten/methods/definitions/person-with-a-western-migration-background. Accessed Jul 2021]

STARSHIP STUDY GROUP

Martin G.A. Baartmans[8], Jolita Bekhof[9], Harry Buijs[10,11], Jan Erik Bunt[12], Peter H. Dijk[13], Christian V. Hulzebos[13], Ralph W.J. Leunissen[14], Ben J.P.W. Snoeren[15], Bente de Vries[16], Leo Wewerinke[17].

[8]Department of Paediatrics, Maasstad Hospital, Rotterdam, the Netherlands.

[9]Department of Paediatrics, Isala – Amalia Children's Clinic, Zwolle, the Netherlands.

[10]Primary care birth centre Haga, The Hague, the Netherlands.

[11]Primary care birth centre Maasstad, Rotterdam, the Netherlands.

[12]Department of Paediatrics, Elisabeth-TweeSteden Hospital, Tilburg, the Netherlands.

[13]Department of Neonatology, University Medical Centre Groningen – Beatrix Children's Hospital, University of Groningen, Groningen, the Netherlands.

[14]Department of Paediatrics, Haaglanden Medical Centre Westeinde, The Hague, the Netherlands.

[15]Primary care birth centre Fam, Tilburg, the Netherlands.

[16]Primary care birth centre Westeinde, The Hague, the Netherlands.

[17]Department of Paediatrics, Haga Hospital – Juliana Children's Hospital, The Hague, the Netherlands.
